# Atypical Presentation of Perforated Viscus as Biliary Colic

**DOI:** 10.7759/cureus.12513

**Published:** 2021-01-05

**Authors:** Abdullah M Almuebid, Zainab Y Alsadah, Hussain Al Qattan, Abdullah A Al Mulhim, Dunya Alfaraj

**Affiliations:** 1 Emergency Medicine, King Fahad University Hospital, Dammam, SAU; 2 Emergency Medicine, King Fahad University Hospital, Alkhobar, SAU; 3 Internal Medicine, King Fahad University Hospital, Dammam, SAU; 4 Emergency Medicine, King Fahad Hospital of the University, Alkhobar, SAU; 5 Emergency Medicine, Imam Abdulrahman Bin Faisal University, King Fahad University Hospital, Dammam, SAU

**Keywords:** peptic ulcer disease, perforated duodenal ulcer, peritonitis

## Abstract

Peptic ulcer is a defect in the mucosal layer of the stomach or duodenum that extends into the deeper layers of their walls. Patients with peptic ulcer disease (PUD) may be asymptomatic or have mild abdominal discomfort. It is one of the common etiologies of perforated viscus resulting in secondary peritonitis, a life-threatening condition that carries high risk for morbidity and mortality especially in those who present late to the hospital or due to unrecognized and misdiagnosed perforation. Early detection of perforation of peptic ulcers should be based on clinical data and imaging techniques. We report a case of a 56-year-old female who presented to our ED with right upper quadrant (RUQ) pain radiating to the right shoulder, alleviated by food, and not aggravated by anything. On examination, the patient was vitally stable, tenderness in the RUQ was appreciated, and Murphy sign was positive. Thus, she was diagnosed with perforation of anterior first part of the duodenum. What makes our case peculiar is the presentation of biliary colic in the setting of perforated peptic ulcer.

## Introduction

Peptic ulcer disease (PUD) is described as an interruption of the inner lining of the gastrointestinal (GI) tract due to excessive gastric acid secretion or pepsin. This defect can extend to the deeper layers of the GI tract reaching up to the muscularis propria. Ordinarily it involves the stomach and/or proximal duodenum, however, it could involve lower esophagus, distal duodenum, or even jejunum [1]. Patients with PUD can present with a wide range of clinical features ranging from being completely asymptomatic or having mild abdominal discomfort to life-threatening complications such as perforated PUD [2]. It is estimated that the annual incidence of perforated PUD is 3.8-14 per 100000 individuals and the 30-day morality is 8.6%. Several factors were associated with higher mortality including advanced age, co-morbidities, shock, and treatment delay [3]. Therefore, immediate and early identification of patients with perforated PUD is critical and favors a better prognosis. Classical symptoms of perforated PUD are epigastric abdominal pain, tenderness and rigidity upon examination and vital signs instability. Nevertheless, it is crucial to beware of atypical presentations of perforated PUD to ensure early treatment [2]. We are reporting a case of perforated duodenal ulcer in which the patient presented with typical biliary colic pain and upon further investigations biliary pathologies were ruled out and perforated PUD was confirmed.

## Case presentation

**Figure 1:**
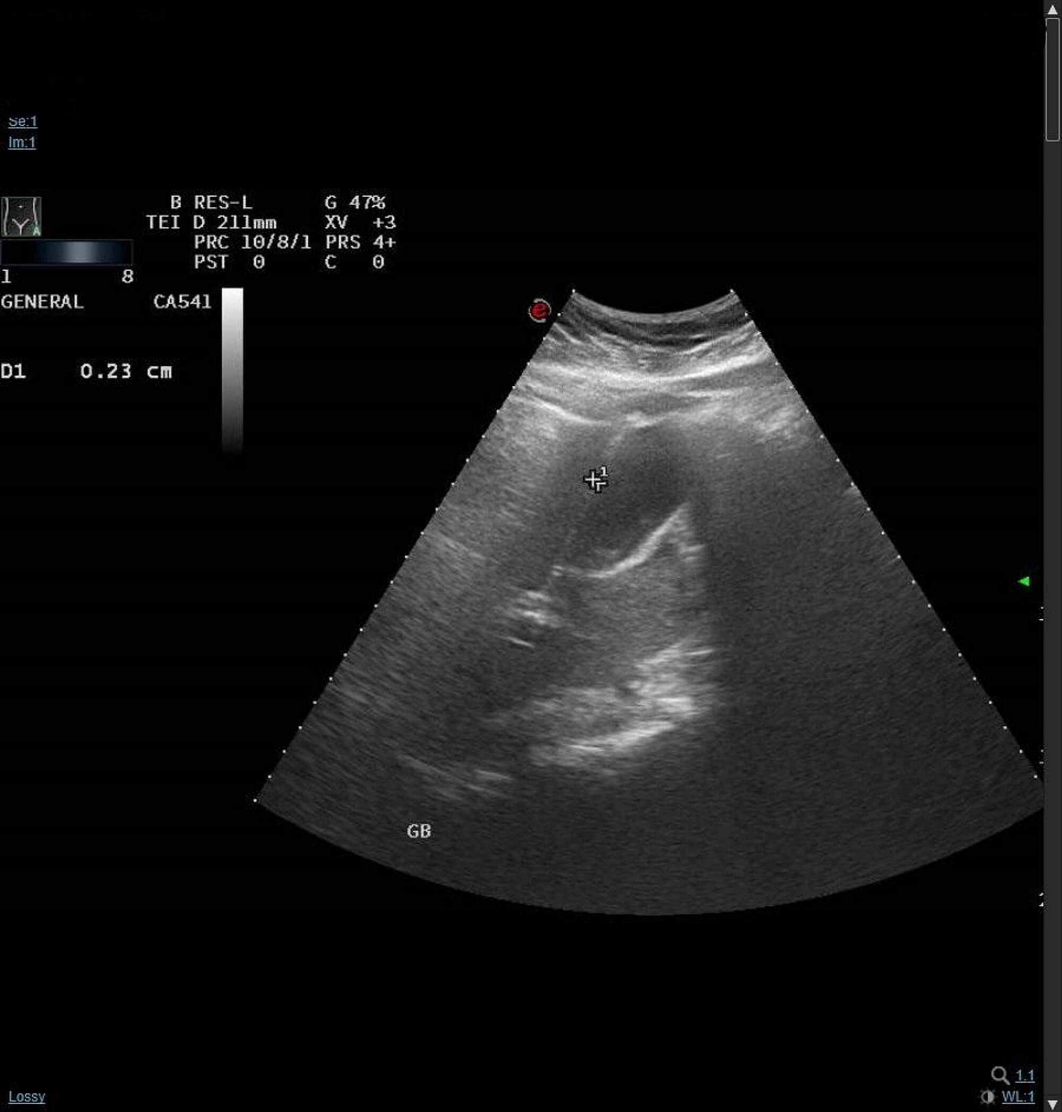
Ultrasound of gallbladder showing no significant changes.

A 56-year-old female had a known case of hypertension, dyslipidemia, and epilepsy. She presented to our ED complaining of right upper quadrant (RUQ) abdominal pain for two weeks. The pain was burning in nature, radiating to the right shoulder, alleviated by food, and no aggravating factors. The onset of pain was insidious and intermittent, but in the last two days it progressed to be severe enough to wake her up from sleep. It was associated with constipation which was relieved by laxatives. The patient had a history of non-steroidal anti-inflammatory drugs (NSAID) consumption for six months for her knee pain caused by osteoarthritis. She denied any history of nausea, vomiting, hematemesis, hematochezia, melena, fever, or jaundice. Upon examination, the patient appeared in pain, but no jaundice or pallor was noted. Vital signs were as following: blood pressure 150/81 mmHg, temperature 36.6 degree celsius, pulse 67 beats/min, respiratory rate 20 breaths/min, and O2 saturation 100 on room air. On palpation of the abdomen, it was soft and lax with tenderness in the RUQ and positive Murphy sign. Other systemic examinations were insignificant. The following investigations were ordered: complete blood count, renal function test, liver function test, lactate dehydrogenase (LDH), lactic acid, lipase, venous blood gas, and electrocardiogram. All the tests were normal. Then ultrasound of abdomen was performed, it showed gallbladder calculi with no signs of cholecystitis and there was no free peritoneal fluid (Figure 1).

**Figure 2:**
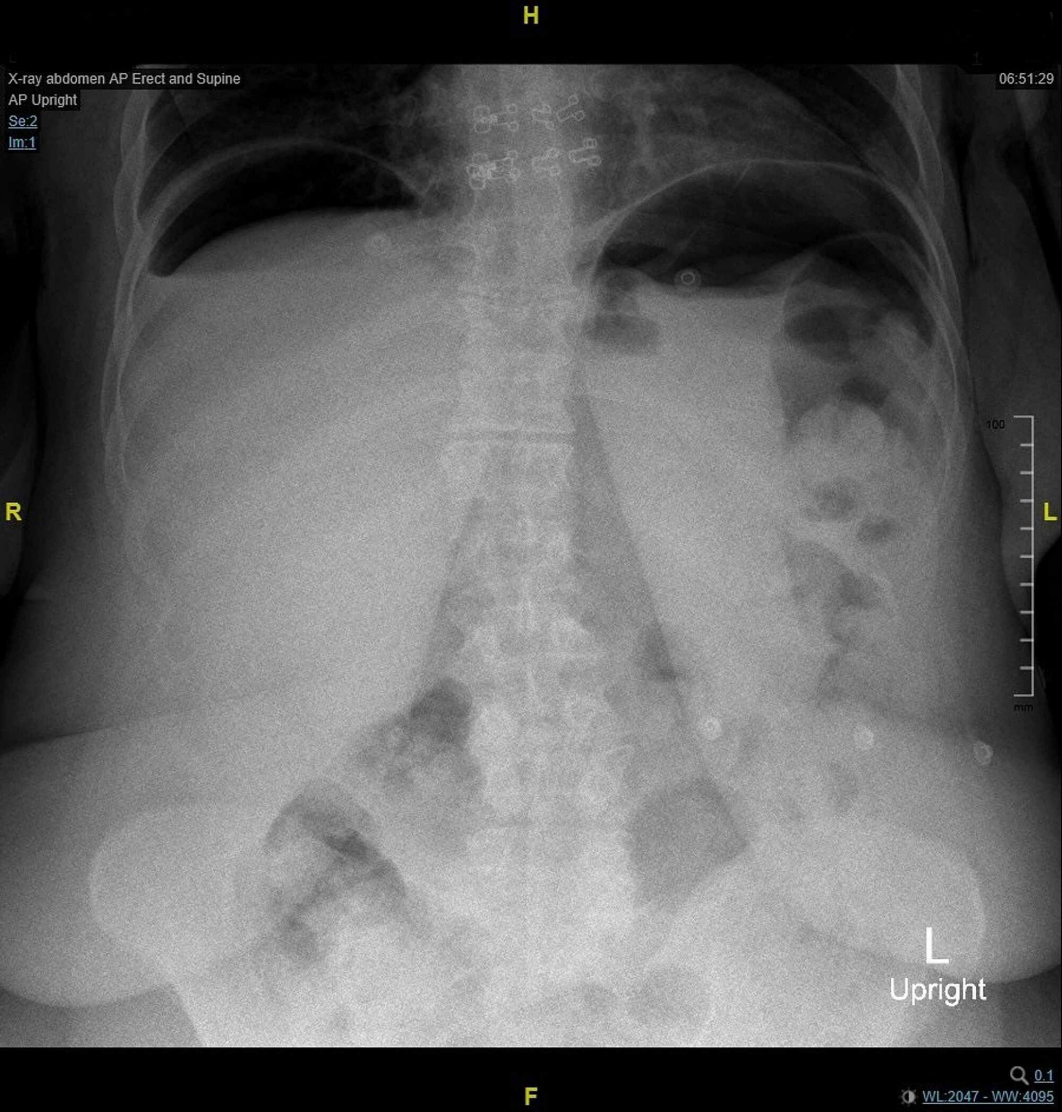
Erect abdominal X-ray showing air under diaphragm.

In addition, supine abdominal and chest X-rays were done and results were unremarkable. Initially, the patient refused to do an erect abdominal X-ray. Ultimately, it was done after receiving 5 mg of morphine that improved her pain. The result showed air under the diaphragm (Figure 2) and CT scan confirmed the presence of a perforated distal stomach with pneumoperitoneum and free fluid (Video 1).

 

**Video 1:** Abdominal CT scan showing perforated duodenum with pneumoperitoneum and free fluid.

General surgery was consulted, and they decided to perform an exploratory laparotomy. During the operation, a small perforation in the anterior first part of the duodenum was identified, and washout and simple Graham patch repair was done. She tolerated the procedure well without any complications. She shifted to the recovery room in good condition and was discharged on the third day of admission.

## Discussion

Perforated viscus is one of the most challenging diagnoses in the setting of acute abdomen presentation. A broad spectrum of diseases such as gastroenteritis, PUD, ischemic colitis, inflammatory bowel disease, diverticulitis, and appendicitis that present with clear manifestations can increase the suspicion of perforated hollow viscus which eventually leads to secondary peritonitis. Focused and clear history, complete physical examination, relevant radiographic and laboratory studies are essential to prompt the diagnosis [4]. The presentation of generalized abdominal pain and tenderness, board-like abdominal wall rigidity, and hypoactive bowel sounds may be covert in older patients or those on certain medications such as steroids, immunosuppressants, and/or narcotic analgesics, in addition to obesity, difficulty in communication, and previous abdominal surgery [5-6].

At the initial evaluation, vital signs and laboratory tests should be promptly assessed. Also, signs of systemic inflammatory response syndrome and septic shock can expedite the need for resuscitation and interventional measures [7]. Abdominal ultrasound and conventional plain X-rays are routinely considered as the initial part of workup in patients with acute abdomen. This approach can be useful to identify the presence of a hollow viscus perforation and the underlying etiology. Perforation and leakage can be detected on ultrasound in which is more likely to determine the underlying cause of pain than a plain-film radiograph. While the presence of pneumoperitoneum on plain abdominal X-ray can confirm the diagnosis particularly at the late stage without localizing the perforation exact site [8]. However, CT of the abdomen and pelvis is the most sensitive and specific test to diagnose and confirm a perforation and ascertain the most likely etiology [7].

Peptic ulcer disease is one of the most common causes of perforation. Although PUD is frequently considered a rather benign disease, however, when complications occur it becomes a life-threatening condition that carries a high risk for morbidity and mortality. With regard to the localization of PUD perforation, it is mostly present in the proximal part of the duodenum in 35%-65% of the cases, 25%-45% are located in the pylorus, and 5%-25% are in the stomach. The most common etiologies are helicobacter pylori infection and chronic use of NSAIDs [9]. Other factors include excessive stress, smoking, and consumption of alcohol or coffee. The definitive management is surgery by simple closure with omental patch [10].

Ambiguous clinical features in those with small perforation of the stomach and duodenum lead to misdiagnosis that in return delay the management putting patients in potential risk of complications and prolonged recovery duration [7]. Nonspecific clinical presentations were reported in several cases. In one case, a 45-year-old male presented with left upper quadrant pain, vomiting, and fever. Abdominal examination revealed restricted abdominal movement on the left side with moderate distension and tenderness in the epigastric area but maximally on the left upper quadrant. The right side was completely free from distension, tenderness, or rigidity. Systematic exploration revealed a left subphrenic abscess secondary to perforated duodenal ulcer [11]. In another case, a 35-year-old female with a known case of PUD presented with history of epigastric pain and nausea for four hours. On examination, her vital signs were normal except for mild tachycardia with mild tenderness and garding in the upper abdomen. Regarding laboratory tests and imaging, they were insignificant. On the second day of admission, the patient deteriorated and ultrasound was done which revealed free fluid in the abdomen. As a result, emergency laparotomy was done revealing perforation in the anterior wall of the proximal part of the duodenum with live Ascaris worm coming out of it [12].

Furthermore, another study reported four cases of fatality due to atypical presentations of perforated PUD. One of the cases reported is a 37-year-old male known to be an ethanol abuser who presented to the ED with two days history of abdominal pain, nausea, vomiting, and diarrhea. Upon evaluation, he was vitally stable with a soft abdomen, normoactive bowel sound, mild leukocytosis, hypokalemia, and normal imaging. He was discharged as a case of gastroenteritis. After 11 hours the patient was found dead at home, and the autopsy showed a perforated duodenal ulcer [13].

With regard to our patient, she presented with RUQ pain radiating to her shoulder, and she was hemodynamically stable. On examination Murphy’s sign was positive, and laboratory and imaging studies initially were insignificant. This presentation increased the suspicion of biliary colic so the patient would be discharged after ruling out acute cholecystitis and if the pain improved. Due to the fact that our patient was hemodynamically stable and presenting as a case of biliary colic it made such presentation a challenging one to reach to a clear diagnosis of perforated duodenal ulcer. 

## Conclusions

Perforated PUD may present with a wide variety of nonspecific symptoms which makes early diagnosis a difficult task to be established. Moreover, it is a potentially fatal condition that carries out high risk of mortality if treatment was delayed, which justify the importance of high index of suspicion to identify patient at risk early in the course of the disease and to provide necessary intervention.
